# Data processing pipeline for cardiogenic shock prediction using machine learning

**DOI:** 10.3389/fcvm.2023.1132680

**Published:** 2023-03-23

**Authors:** Nikola Jajcay, Branislav Bezak, Amitai Segev, Shlomi Matetzky, Jana Jankova, Michael Spartalis, Mohammad El Tahlawi, Federico Guerra, Julian Friebel, Tharusan Thevathasan, Imrich Berta, Leo Pölzl, Felix Nägele, Edita Pogran, F. Aaysha Cader, Milana Jarakovic, Can Gollmann-Tepeköylü, Marta Kollarova, Katarina Petrikova, Otilia Tica, Konstantin A. Krychtiuk, Guido Tavazzi, Carsten Skurk, Kurt Huber, Allan Böhm

**Affiliations:** ^1^ Premedix Academy, Bratislava, Slovakia; ^2^ Department of Complex Systems, Institute of Computer Science, Czech Academy of Sciences, Prague, Czech Republic; ^3^ Clinic of Cardiac Surgery, National Institute of Cardiovascular Diseases, Bratislava, Slovakia; ^4^ Faculty of Medicine, Comenius University in Bratislava, Bratislava, Slovakia; ^5^ The Leviev Cardiothoracic & Vascular Center, Chaim Sheba Medical Center, Ramat Gan, Israel; ^6^ Affiliated to the Sackler Faculty of Medicine, Tel Aviv University, Tel Aviv, Israel; ^7^ 3rd Department of Cardiology, National and Kapodistrian University of Athens, Athens, Greece; ^8^ Global Clinical Scholars Research Training (GCSRT) Program, Harvard Medical School, Boston, MA, United States; ^9^ Department of Cardiology, Faculty of Human Medicine, Zagazig University, Zagazig, Egypt; ^10^ Cardiology and Arrhythmology Clinic, Marche Polytechnic University, University Hospital “Umberto I - Lancisi - Salesi”, Ancona, Italy; ^11^ Department of Cardiology Angiology and Intensive Care Medicine, Deutsches Herzzentrum der Charité (DHZC), Campus Benjamin Franklin, Charité - Universitätsmedizin Berlin, Berlin, Germany; ^12^ Berlin Institute of Health, Charité—Universitätsmedizin Berlin, Berlin, Germany; ^13^ Deutsches Zentrum für Herz-Kreislauf-Forschung e.V., Berlin, Germany; ^14^ Institute of Medical Informatics, Charité—Universitätsmedizin Berlin, Berlin, Germany; ^15^ Department for Cardiac Surgery, Cardiac Regeneration Research, Medical University of Innsbruck, Innsbruck, Austria; ^16^ 3rd Medical Department, Cardiology and Intensive Care Medicine, Wilhelminen Hospital, Vienna, Austria; ^17^ Department of Cardiology, Ibrahim Cardiac Hospital & Research Institute, Dhaka, Bangladesh; ^18^ Cardiac Intensive Care Unit, Institute for Cardiovascular Diseases of Vojvodina, Sremska Kamenica, Serbia; ^19^ Faculty of Medicine, University of Novi Sad, Novi Sad, Serbia; ^20^ Cardiology Department, Emergency County Clinical Hospital of Oradea, Oradea, Romania; ^21^ Institute of Cardiovascular Sciences, University of Birmingham, Medical School, Birmingham, United Kingdom; ^22^ Department of Internal Medicine II, Division of Cardiology, Medical University of Vienna, Vienna, Austria; ^23^ Duke Clinical Research Institute Durham, NC, United States; ^24^ Department of Clinical-Surgical, Diagnostic and Paediatric Sciences, University of Pavia, Pavia, Italy; ^25^ Anesthesia and Intensive Care, Fondazione Policlinico San Matteo Hospital IRCCS, Pavia, Italy; ^26^ Department of Acute Cardiology, National Institute of Cardiovascular Diseases, Bratislava, Slovakia

**Keywords:** classification, machine learning, missing data imputation, processing pipeline, prediction model, cardiogenic shock

## Abstract

**Introduction:**

Recent advances in machine learning provide new possibilities to process and analyse observational patient data to predict patient outcomes. In this paper, we introduce a data processing pipeline for cardiogenic shock (CS) prediction from the MIMIC III database of intensive cardiac care unit patients with acute coronary syndrome. The ability to identify high-risk patients could possibly allow taking pre-emptive measures and thus prevent the development of CS.

**Methods:**

We mainly focus on techniques for the imputation of missing data by generating a pipeline for imputation and comparing the performance of various multivariate imputation algorithms, including k-nearest neighbours, two singular value decomposition (SVD)—based methods, and Multiple Imputation by Chained Equations. After imputation, we select the final subjects and variables from the imputed dataset and showcase the performance of the gradient-boosted framework that uses a tree-based classifier for cardiogenic shock prediction.

**Results:**

We achieved good classification performance thanks to data cleaning and imputation (cross-validated mean area under the curve 0.805) without hyperparameter optimization.

**Conclusion:**

We believe our pre-processing pipeline would prove helpful also for other classification and regression experiments.

## 1. Introduction

Modern technology, increasing computing power, and advances in machine learning provide new possibilities to process and extract maximum knowledge from available patient data that can improve healthcare, patient outcomes (1–4), and new frontiers in predictive medicine (5).

The MIMIC dataset (Medical Information Mart for Intensive Care) (6) is a widely-used public data source including over fifty thousand de-identified electronic health records (EHR) of patients admitted to critical care units at Beth Israel Deaconess Medical Center in Boston, MA, the USA, from 2001 to 2012. This database contains a large amount of clinical data, which resulted in several analytic studies in cardiovascular medicine (7–9).

Unfortunately, the data analysis requires a cautious pre-analytic phase of meticulous data cleaning and processing which may be particularly challenging in multi-national observational studies and registries (10). In this paper, we describe in detail our methodology for processing the MIMIC dataset as a part of developing a scoring system for predicting cardiogenic shock (CS) in patients suffering from acute coronary syndrome (ACS) (11).

Despite improvements in diagnostic and treatment options, CS still affects 10% of ACS patients with unacceptably high, reaching nearly 50% mortality (12). CS is not only an isolated decrease in cardiac function but a rapidly progressing multiorgan dysfunction accompanied by severe cellular and metabolic abnormalities, and when developed, even the elimination of the underlying primary cause is not to reverse this vicious circle (13). The aim of the STOPSHOCK project is to derivate and validate a simple scoring system able to identify high-risk patients prior to the development of CS. Such patient stratification could allow us to take pre-emptive measures, such as the implantation of percutaneous mechanical circulatory support, and thus prevent the development of CS, ultimately leading to improved survival of ACS patients.

## 2. Methods

### 2.1. First cohort selection

In our study, we included patients presenting with ACS undergoing cardiac catheterization. The cohort was then divided into two groups: a patient group, comprising patients who developed cardiogenic shock during hospitalization, and a control group, comprising patients who did not develop cardiogenic shock. The patients were selected and assigned to a cohort based on the diagnosis and procedures undertaken during the hospitalization.

The identification of diagnosis and management was made using the ICD9 coding scheme (14).

The ICD9 codes for both cohorts are detailed in Table 1. Briefly, the control group included patients with acute myocardial infarction, ischemic heart disease, and angina pectoris undertaking cardiac catheterization, but excluded cardiogenic or unspecified shock. Conversely, the patient group contained patients who developed cardiogenic shock, in addition to the myocardial infarction diagnoses and catheterization codes.

**Table 1:** Initial cohort selection based on ICD9-coded diagnoses and procedures.

ICD9 diagnosis	title	patients	controls
78551	Cardiogenic shock	x	
78550	Shock, unspecified	x	
41000–41092	Various versions of Acute myocardial infarction	x	x
41189	Other acute and subacute forms of ischemic heart disease, other	x	x
4139	Other and unspecified angina pectoris	x	x
ICD9 procedures	title	patients	controls
0066	Percutaneous transluminal coronary angioplasty (PTCA)	x	x
3604	Intracoronary artery thrombolytic infusion	x	x
3606	Insertion of non-drug-eluting coronary artery stent(s)	x	x
3607	Insertion of drug-eluting coronary artery stent(s)	x	x
3609	Other removal of coronary artery obstruction	x	x
8855	Coronary arteriography using a single catheter	x	x
8856	Coronary arteriography using two catheters	x	x
8857	Other and unspecified coronary arteriography	x	x
3722	Left heart cardiac catheterization	x	x
3723	Combined right and left heart cardiac catheterization	x	x
total *n*.		703	3056

ICD, international classification of diseases.

The final number of unique hospital stays for our control group reached 3,056, while we included 703 hospital stays for the patient group.

However, based on coded data, it was not possible to reliably distinguish between patients who were already admitted with shock and those who developed shock during hospitalization.

Several methods were tested based on the variation of patient variables (blood pressure, heart rate, use of inotropes, fluid replacement therapy, or similar) (15, 16). However, none of these methods provided reliable results when verified based on textual hospitalization summaries.

Ultimately, we analyzed individual discharge reports provided within the dataset. A total of 703 summaries were manually sorted, which resulted in 172 unique hospital admissions of patients who developed cardiogenic shock during the hospital course.

### 2.2. Data inspection

All available data were inspected, plotted, and sorted based on missing values. Potentially relevant clinical variables to the aim of the study were selected. As several variables are stored in the database using multiple codes for the same variable, the ones selected were clustered into aggregated variables. For example, systolic blood pressure is available as *Non-Invasive Blood Pressure systolic, Arterial BP [Systolic], Manual Blood Pressure Systolic Left, Manual Blood Pressure Systolic Right, Arterial Blood Pressure systolic, ART BP Systolic, Manual BP [Systolic].* In our case this clustering concerned mean, systolic and diastolic arterial pressures. For this scoring system, the first recorded variables were selected.

When different units of measure were used, they were converted to the international standard. Outliers were inspected manually. Some values were manually corrected (e.g., 375 °C to 37.5 °C). Extremal values above the common threshold—clearly incorrectly entered values (e.g., the body temperature of 5 °C) were deleted.

**Figure 1:**
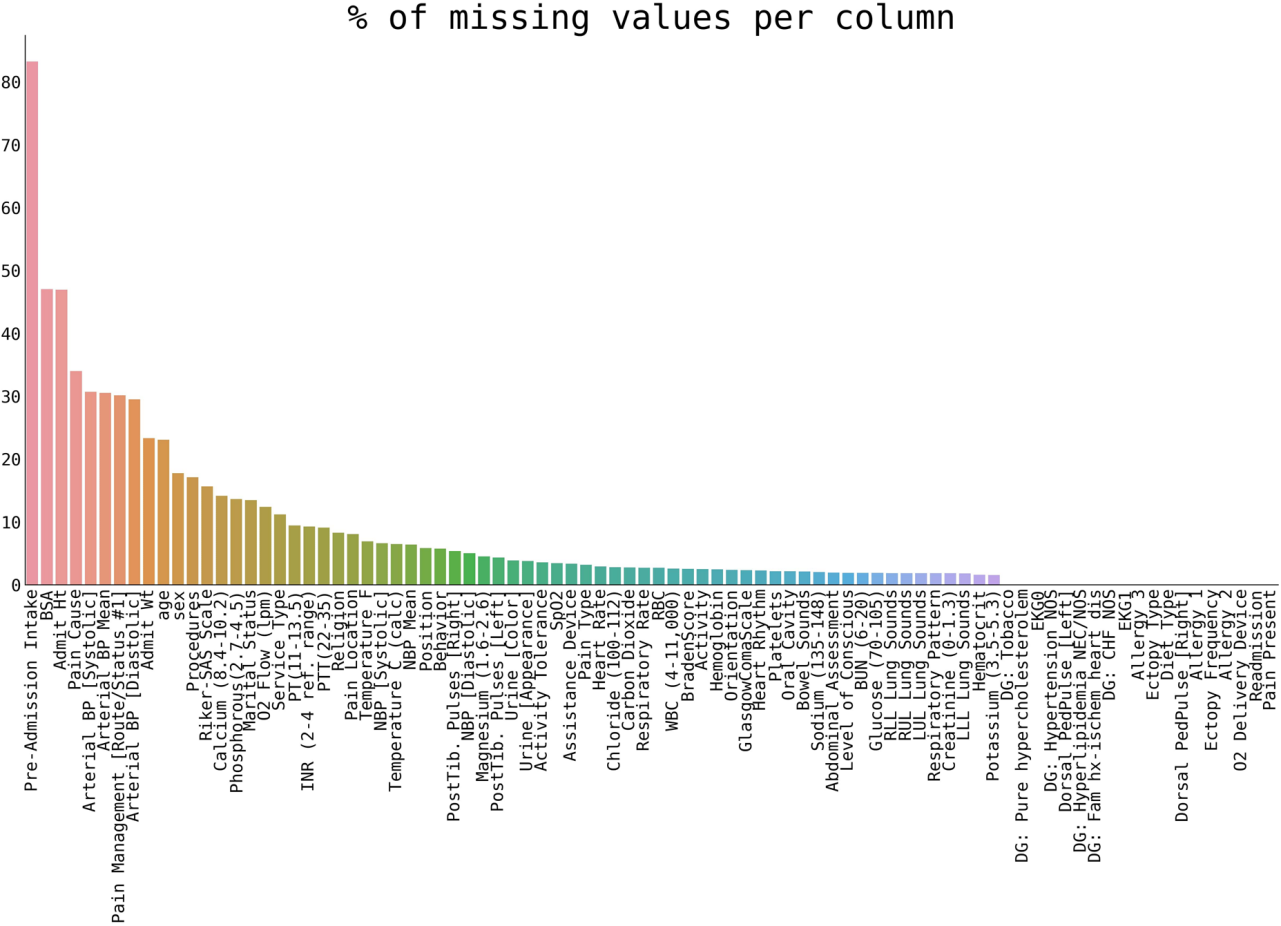
The percentage of missing values per variable in our selection of patients.

In the preselected 84 variables, 7.86% of missing values were found (Figure 1) and the missing data were missing completely at random (17).

### 2.3. Pre-imputation cohort and variables selection

In order to improve the predictive ability of the scoring system, missing values of preselected features were imputed. Many different univariate (18, 19) and multivariate techniques (18, 20) have been described for data imputation. We used multivariate techniques considering the rate of missing values that were missing completely at random and the relatively high number of variables. Additionally, multivariate imputation techniques can accommodate and mimic interdependencies between variables (21), which seemed more appropriate for the current study. To improve the predictive value of multivariate imputation techniques, we decided to enlarge our initial groups, include more variables, and expand our original dataset both in terms of patients and variables included.

We merged patient and control groups into one cohort for the imputation. Next, we included similar patients by reducing the inclusion criteria to:

Patients with at least one ICD9 diagnosis code:

78551: Cardiogenic shock
78550: Shock, unspecified
41000–41092: various versions of Acute myocardial infarction
41189: Other acute and subacute forms of ischemic heart disease, other
4139: Other and unspecified angina pectoris

The advantage of selecting patients with a related diagnosis compared to random selection is a greater similarity of data, which should theoretically result in higher imputation accuracy (22).

As for additional variables used for the sole purpose of missing data imputation, we included 19 clinically relevant additional variables with minimal missing values. These 19 additional variables are detailed in Table 2. The final pre-imputation dataset contained 4,595 patients in one grouped cohort and 86 variables.

**Table 2:** Overview of the variables from the MIMIC III database that were used as a pre-imputation dataset.

variable	missing values (%)	variable	missing values (%)
Pre-Admission Intake	83.2	RBC	2.68
BSA	46.99	WBC (4–11,000)	2.55
Admit Hit	46.92	Braden Score	2.52
Pain Cause	33.97	Activity	2.46
Arterial BP [Systolic]	30.66	Hemoglobin	2.44
Arterial BP Mean	30.49	Orientation	2.33
Pain Management [Route/Status #1]	30.1	Glasgow Coma Scale	2.29
Arterial BP [Diastolic]	29.49	Heart Rhythm	2.24
Admit Wt	23.31	Platelets	2.15
age	23.5	Oral Cavity	2.15
sex	17.74	Bowel Sounds	2.11
Procedures	17.8	Sodium (135–148)	2.0
Riker-SAS Scale	15.63	Abdominal Assessment	1.92
Calcium (8.4–10.2)	14.15	Level of Conscious	1.89
Phosphorous (2.7–4.5)	13.6	BUN (6–20)	1.87
Marital Status	13.45	Glucose (70–105)	1.87
O2 Flow (lpm)	12.38	RLL Lung Sounds	1.85
Service Type	11.19	RUL Lung Sounds	1.85
PT (11–13.5)	9.42	LUL Lung Sounds	1.83
INR (2–4 ref. range)	9.23	Respiratory Pattern	1.83
PTT (22–35)	9.8	Creatinine (0–1.3)	1.83
Religion	8.27	LLL Lung Sounds	1.81
Pain Location	8.3	Hematocrit	1.57
Temperature F	6.88	Potassium (3.5–5.3)	1.55
NBP [Systolic]	6.59	DG: Tobacco	0.0
Temperature C	6.46	DG: Pure hypercholesterolem	0.0
NBP Mean	6.38	EKG0	0.0
Position	5.81	DG: Hypertension NOS	0.0
Behavior	5.72	Dorsal PedPulse [Left]	0.0
PostTib. Pulses [Right]	5.35	DG: Hyperlipidemia NEC/NOS	0.0
NBP [Diastolic]	4.98	DG: Fam hx-ischem heart dis	0.0
Magnesium (1.6–2.6)	4.48	DG: CHF NOS	0.0
PostTib. Pulses [Left]	4.31	EKG	0.0
Urine [Color]	3.58	Allergy 3	0.0
Urine [Appearance]	3.74	Ectopy Type	0.0
Activity Tolerance	3.55	Diet Type	0.0
SpO2	3.44	Dorsal PedPulse [Right]	0.0
Assistance Device	3.33	Allergy 1	0.0
Pain Type	3.18	Ectopy Frequency	0.0
Heart Rate	2.89	Allergy	0.0
Chloride (100–112)	2.76	O2 Delivery Device	0.0
Carbon Dioxide	2.72	Readmission	0.0
Respiratory Rate	2.7	Pain Present	0.0

The table shows the percentage of missing values per variable in our selection of 4595 patients.

### 2.4. Data imputation

We utilized Multiple Imputation by Chained Equations (MICE) (23–25) as our primary algorithm for the missing data imputation. The MICE algorithm imputes missing data through an iterative series of predictive models. In each iteration, specified variables in the dataset are imputed using other variables. These iterations are run until it appears that convergence has been met. Gradient-boosted, tree-based predictive models were implemented as a part of the *LightGBM* package (26). Moreover, the predictive mean matching technique (PMM) was also used during the imputation (27). PMM entails the selection of a data point from the original, non-missing data with a predicted value close to the predicted value of the missing sample. The closest five values are chosen as candidates, from which a value is sampled randomly. By using PMM, we could correctly impute variables with the multimodal empirical distribution. By exploiting the stochastic nature of tree-based predictive models, we could impute multiple versions of the dataset. This allowed us to run a sensitivity analysis and assess the effect of missing data on our final classification model. As a good balance between computational time and statistical power, we decided to run the imputation ten times. In order to have a benchmark for our stability analysis, we further selected three additional imputation algorithms: k-Nearest Neighbors (KNN) (28), “Soft Impute” (performs matrix completion by iterative soft thresholding of SVD decomposition) (29), and “Iterative SVD” (performs matrix completion by iterative low-rank SVD decomposition) (30).

### 2.5. Final cohort and variable selection

After successfully imputing all 13 datasets (10 with MICE, 1 with KNN, 1 with Soft Impute, and 1 with Iterative SVD), the final selection of the control and patient group, and variables to be used for the diagnostic model was made. Here, we used our initial selection and discarded added patients and variables.

### 2.6. Computational methods

All analyses were performed in *python* version 3.8.13 (https://www.python.org) with appropriate packages (*pandas* 1.4.2, *scipy* 1.8.0, *pingouin* 0.5.1, *miceforest* 5.4.0, *lightgbm* 3.3.2, *seaborn* 0.11.2). The repository containing the analysis code will be available after the finalization of this study or upon reasonable request.

## 3. Results

### 3.1. Imputed final dataset statistics

As the first step, the percentage of missing values of preselected variables was plotted (cf. section Pre-imputation cohort and variables selection). The results are shown in Figure 1.

**Figure 2:**
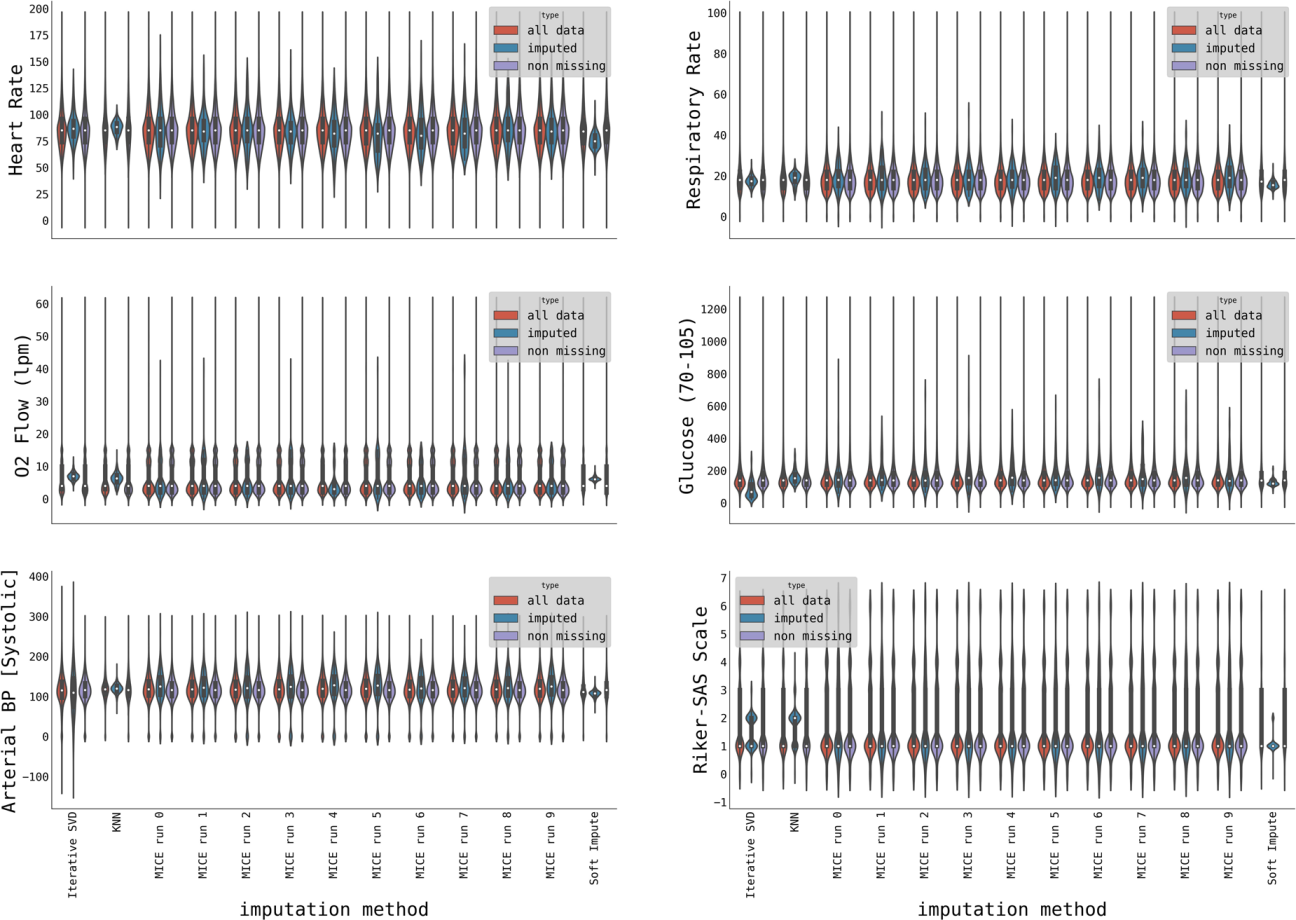
Violin plots showing distributions of initially non-missing data (purple), imputed data (blue), and all data (red) for three imputation methods (with the MICE method, we imputed ten datasets). The distributions are shown for a selection of variables: Heart rate, Respiratory rate, O_2_ flow [lpm], Glucose, Arterial Systolic Blood Pressure, and Riker-SAS scale.

Evaluating the imputation quality is not straightforward, and universally accepted pipelines do not exist (31). We opted for visual assessment to qualitatively estimate the quality of imputation and compare distributions of imputed data with original, non-missing data employing the Kolmogorov-Smirnov test (32) for distribution equivalent for the quantitative assessment. The example of imputation quality for selected variables is shown in Figure 2.

The MICE imputation algorithm correctly captured the data distribution in most cases (Figure 2) including multimodal distribution (e.g., O_2_ flow) differently from other imputation techniques, such as Iterative SVD, KNN, or Soft Impute.

**Figure 3:**
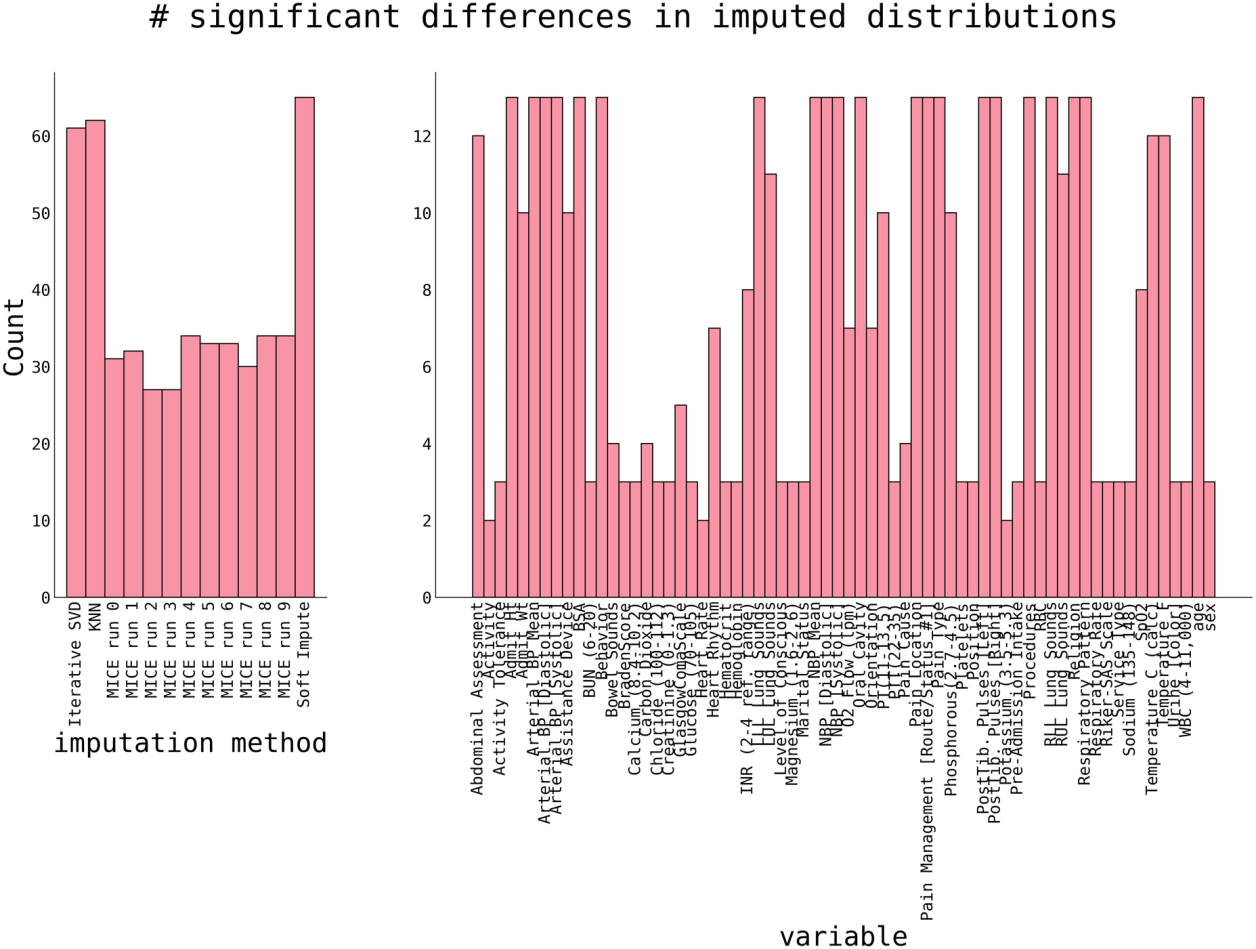
The number of significant differences between original non-missing data and imputed data. Significance was estimated using a 2-sample Kolmogorov-Smirnoff test with *p* < 0.05. *P*-values are corrected for multiple comparisons using a Benjamini-Yekutieli FDR procedure (32). Shown are counts of significant differences per dataset (left panel) and variable (right panel).

The quality of imputation on the whole dataset was performed by comparing the distributions demonstrating approximately 20 significant differences between the original and imputed datasets. At the same time, other methods exhibit almost twice as many (Figure 3).

The right panel of Figure 3 shows the number of significant differences per variable in all imputation methods. Variables with a high number are “hard to impute”. Naturally, this correlates with the percentage of initially missing data (cf. Figure 1), and categorical variables with many different categories (e.g., Lung Sounds, Hearth Rhythm, Respiratory Pattern, and others) are harder to impute. The ability to correctly impute a variable, as shown in Figure 3, will be considered for a final variable selection.

**Figure 4:**
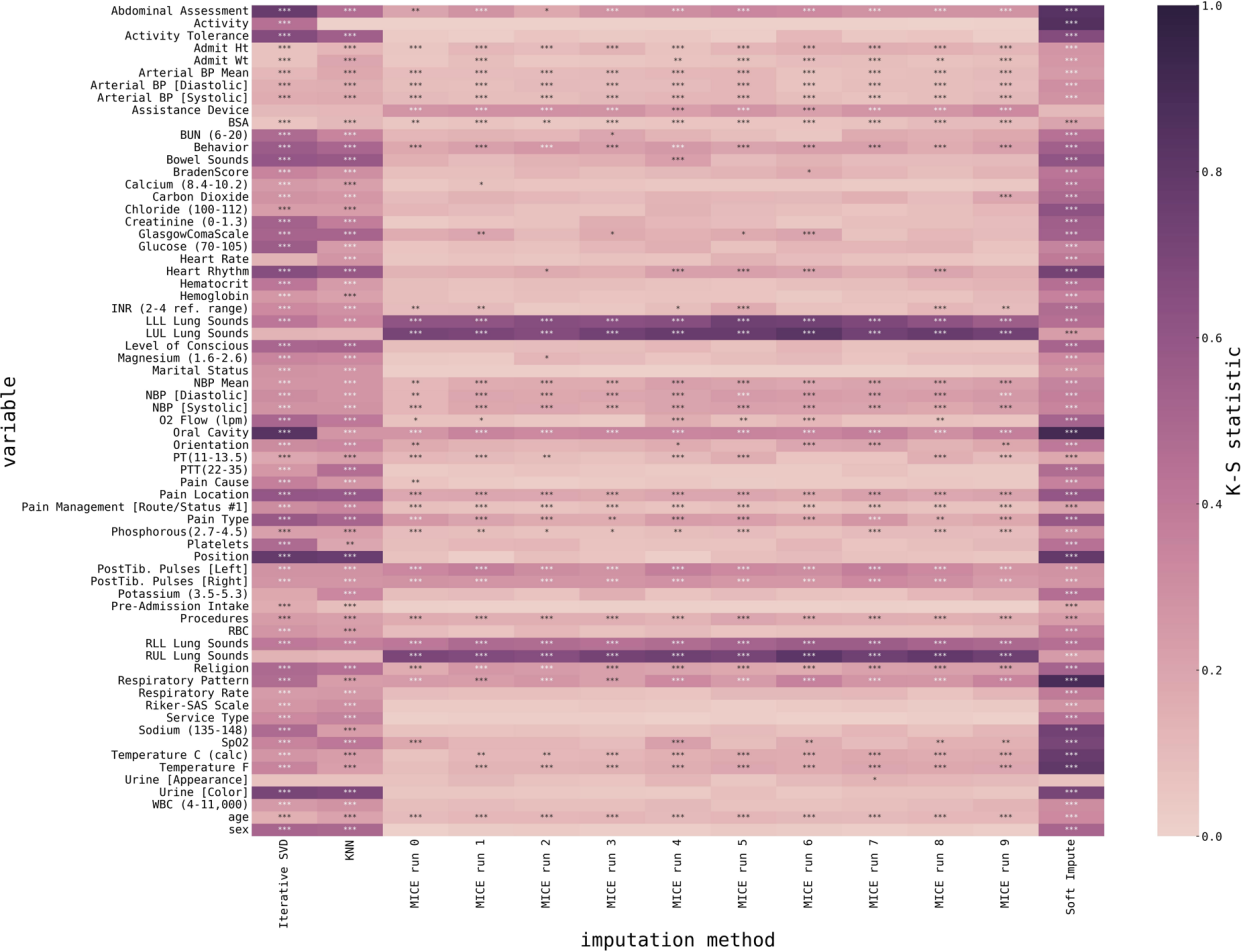
Heatmap representing the match between imputed data and initially non-missing data for all variables and all imputed datasets. Variables are encoded as rows, imputed datasets are encoded as columns, and color encodes the Kolmogorov-Smirnoff statistic as estimated using a 2-sample Kolmogorov-Smirnoff test (lower is better), and stars mark significance (*p* < 0.05 *, *p* < 0.01 **, *p* < 0.001 ***). *P*-values are corrected for multiple comparisons using a Benjamini-Yekutieli FDR procedure (33).

Finally, we also visualized a detailed plot of the ability of our imputation algorithms to estimate the variable distribution, as shown in Figure 4. With very few exceptions, MICE-imputed datasets generally show lower K-S statistics, therefore achieving a better match between imputed and original distribution.

Overall, we observed the superior performance of the MICE method, as for most of the variables, it provides distributions of imputed values closer to the original (i.e., lower Kolmogorov-Smirnoff statistic). However, some variables are better imputed using alternative methods, e.g., Lung Sounds or Assistance Devices.

Numerical variables were all unanimously imputed using the MICE method. Moreover, the variables of medical importance for cardiogenic shock classification (e.g., Heart Rate, O_2_ flow, Glucose, O_2_ saturation) were all imputed using the MICE method with relatively low Kolmogorov-Smirnoff statistic, and *p*-values were in most cases not significant, i.e., we could conclude that MICE imputation provides us with imputed variables that closely resemble original non-missing data.

Our results clearly show the superior ability of the MICE method to reasonably impute data missing completely at random, as in the case of the MIMIC III database. We also suggest imputing more datasets, given the stochastic nature of the imputation. Apart from assessing imputation quality, multiple imputed datasets can be used in later stages for, e.g., sensitivity analysis, in which all datasets are used in grid search for hyperparameter tuning or to increase the number of samples for cross-validation of any diagnostic model. Although an external cohort is critical to validate the model performance of the medical model, cross-validation allows the estimation of the prediction model error. It helps with optimizing the model and classifier selection. Obtaining external medical data for validation is especially difficult due to the sensitive nature and associated protection regulations, so thorough model testing and robust results are usually prerequisites for establishing collaboration.

### 3.2. Cardiogenic shock prediction

We trained a classifier on a subset of 9 clinically relevant variables to test model performance on the imputed dataset. In the first step, we performed a simple bivariate analysis with appropriate statistical tests (chi-squared, unpaired t-test, or Mann-Whitney U-test) for each variable. We chose the ones with a proven or potential pathophysiological connection to cardiogenic shock from the subset of variables with a statistically significant difference. In the next step, we narrowed down the selection to only those variables that are available at the first contact with the patient:

• Heart Rate
• Blood glucose level
• Oxygen saturation
• O_2_ flow of oxygen delivery device
• Arterial blood pressure
• Age
• ECG classification of acute coronary syndrome
• Sex
• History of chronic heart failure

**Figure 5:**
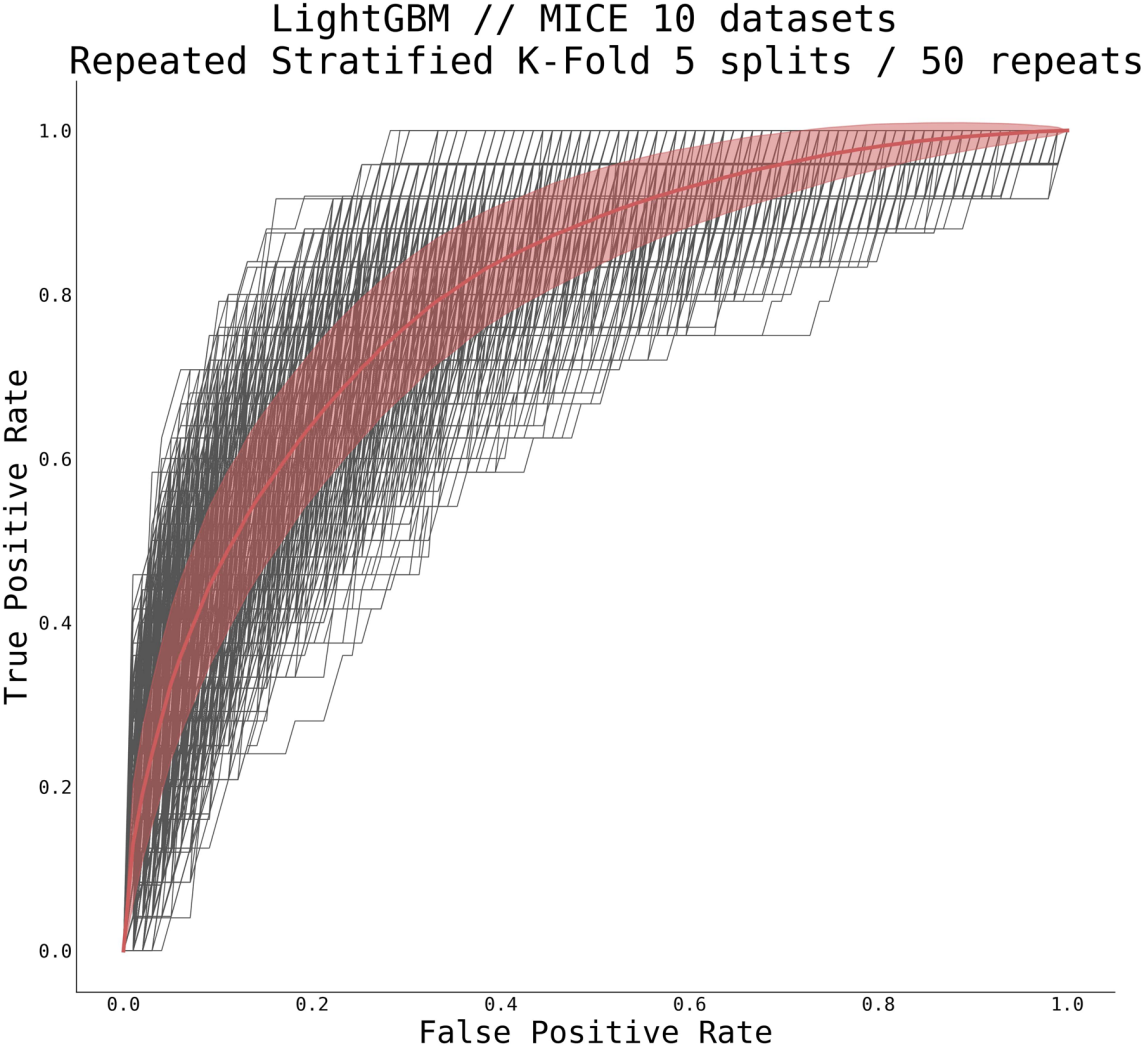
Receiver operating characteristic curve for gradient boosted tree classifier. Shown are all curves from repeated stratified K-Fold cross-validation using five splits and 50 repeats on all 10 MICE-imputed datasets (thin black lines) and mean ± standard deviation over all runs (thick red line). The classifier scored AUC 0.805 ± 0.039.

This cohort consisted of 2,253 patients (123 patients and 2,130 controls). The overview table of statistics in patient and control cohorts is displayed in Table 3. We utilized gradient-boosted trees for the classifier type, representing a strong baseline for these problems. In particular, we utilized a *LightGBM* (26) implementation in *python* with traditional gradient-boosted decision trees and 100 estimators, each using 31 leaves with balanced class weight. Due to a relatively low incidence of cardiogenic shock in patients with the ACS (between 5 and 10%) (12), there was a relatively large class imbalance within our cohort (approximately eight times more controls than patients). To compensate for this fact (and after testing various methods and techniques including manually setting class weights, or using solely under- or over-sampling), we used a combination of over- and under-sampling using the Synthetic Minority Over-sampling Technique (SMOTE) algorithm (34) for over-sampling, followed by Edited Nearest Neighbours (35) cleaning, as implemented in the *imblearn python* package (36). The overall performance of our trained model is summarized in Figure 5.

**Table 3:** Summary of datasets.

	no CS	CS	*p*-value
	[*n* = 123]	[*n* = 2130]	
Sex (male)	64.46%	62.3%	0.698
Heart Rhythm			<0.001
Sinus tachycardia	10.27%	28.69%	
Sinus bradycardia	5.3%	1.64%	
Ventricular tachycardia	0.14%	0.82%	
1st degree AV block	0.85%	1.64%	
Other	83.44%	67.21%	
AH hist. (no)	47.47%	65.57%	0.001
chHF hist. (no)	64.32%	32.79%	<0.001
Hypercholest. hist. (no)	72.93%	87.7%	<0.001
EKG			<0.001
Anterior STEMI or LBBB	17.61%	32.79%	
Other STEMI	24.85%	31.15%	
NSTEMI	47.18%	28.69%	
Other	10.36%	7.38%	
Heart Rate [bpm]	82.1 ± 16.343	94.1 ± 18.912	<0.001
Respiratory Rate [bpm]	17.1 ± 5.549	20.1 ± 5.917	<0.001
Saturation—SpO2 [%]	97.8 ± 3.037	96.3 ± 4.699	0.001
Glucose [mg/dl]	157.8 ± 80.429	227.5 ± 135.491	<0.001
Systolic BP [mmHg]	125.6 ± 24.112	108.9 ± 20.538	<0.001
Age [y]	67.0 ± 12.401	71.0 ± 11.997	<0.001
Shock Index	0.69 ± 0.221	0.90 ± 0.285	<0.001

For continuous variables the table shows mean and standard deviation per dataset and per group. For categorical variables, the table shows percentages of each. AH hist., history of arterial hypertension, chHF hist., history of chronic heart failure, Hyperchol. hist., history of hypercholesterolemia, EKG, electrocardiography, BP, blood pressure, CS, cardiogenic shock.

The average AUC for our trained model, as estimated using repeated stratified K-Fold cross-validation technique with five splits and 50 repeats, reached 0.805 ± 0.039 (CI95% 0.739–0.867). The mean accuracy of our trained classifier using the same cross-validation technique reached 0.893 ± 0.014 (95% CI 0.870–0.915). Considering all issues with missing data, class imbalance, and the number of features used, this preliminary result is more than acceptable and serves as a reasonable basis for further improvement. After hyperparameter tuning or using a different classifier, we expect higher AUC and better performance.

## 4. Discussion

Our results provide a methodical pipeline for data pre-processing for use in extensive EHR such as the MIMIC database. Although some processing steps, such as patient selection, are unique to this specific database, general data processing strategies and imputation techniques are applicable in most medical research working with large datasets.

We have used stochastic and non-stochastic imputation methods in our pipeline to handle missing data. We relied on Multiple Imputation by Chained Equations (MICE) (23–25) as our primary imputation algorithm. We included three additional well-established imputation algorithms (KNN, Soft Impute, and Iterative SVD) (37) to benchmark our stability and sensitivity analysis. Multivariate techniques were chosen for their ability to model interdependencies between variables, thus keeping the covariance structure of the dataset. The evidence based on extensive clinical and epidemiological trials is the cornerstone of modern medicine. Although considerable efforts and fidelity are put into preparation, data collection, and processing, but no dataset is perfect, and missing and incomplete data are unavoidable. Despite the potential of missing data to alter and undermine the validity of research results, this problem has often been overlooked in the medical literature (38). The study by Wood et al. (39) demonstrated that the inadequate handling of missing values with consequent impact on research results is a common problem even in top-tier medical journals (including BMJ, JAMA, Lancet, and New England Journal of Medicine. Moreover, this study has shown that only 21% of the 71 trials included a sensitivity analysis to inspect the quality of imputed data.

Evaluating imputation quality is not well defined, and universally accepted pipelines do not exist (31). In our work, we opted for the visual assessment using heatmaps and violin plots to estimate the characteristics of the imputation qualitatively and for comparison of distributions of imputed data and original, non-missing data by means of the Kolmogorov-Smirnov test (32) with correction for multiple comparisons using a Benjamini-Yekutieli FDR procedure (33) for distribution equivalent for the quantitative assessment. In the pipeline we have studied, MICE imputed datasets have shown a superior ability to impute variables with multimodal distribution compared to other methods. This method's stochastic nature allows imputing multiple datasets and inspecting and comparing their variability. Furthermore, testing the performance of diagnostic models derived from multiple imputed datasets gives more robust results thanks to hyperparameter tuning and increased samples for cross-validation.

Another essential step in our imputation pipeline was the expansion of our original dataset by including more patients and variables. EHR include large quantities of data; usually, only a subset of patients is selected for the specific research based on the inclusion and exclusion criteria. Increasing sample size leads to improved model performance. However, including all available variables in the imputation model would significantly increase model complexity leading to a non-linear increase in computational power needed (with a consequent increase in time and resources needed) and may even lead to model overfitting (40). Selecting patients with similar profiles and variables with clinical and pathophysiological relationships to studied outcomes may lead to optimization and improved model performance (22).

In our case, this methodology enabled us to create a model for predicting CS in ACS patients, which would otherwise be impossible due to the number and distribution of missing values. The ability to identify high-risk patients prior to the development of CS could allow to take pre-emptive measures, such as the implantation of percutaneous mechanical circulatory support, and thus prevent the development of CS leading to improved survival. Predictive medicine is the future of healthcare, ultimately leading to improved patient morbidity mortality and cost reduction (41, 42). Analysis of large EHR is key in developing predictive medicine algorithms, so there is definitely an emerging need for effective processing methodology.

We believe this proposed data processing pipeline offers good instructions for analyzing sizeable electronic health records, mainly focusing on managing missing data. Furthermore, it offers good reproducibility and promotes further research using different cohorts.

## 5. Limitations

Our pipeline was not tested on other datasets. Therefore, the performance might differ in other EHR. Models were selected based on available literature and team experience. Superior computational power would allow imputing and analyzing more datasets and include more models for analysis.

## 6. Conclusion

This proposed data processing pipeline offers good instructions for analyzing sizeable EHR, mainly focusing on managing missing data. Appropriate pre-processing with emphasis on handling of missing data is crucial in analyzing large EHR.

## Data Availability

The raw data supporting the conclusions of this article will be made available by the authors, without undue reservation.
